# Regulatory T cells protect against brain damage by alleviating inflammatory response in neuromyelitis optica spectrum disorder

**DOI:** 10.1186/s12974-021-02266-0

**Published:** 2021-09-15

**Authors:** Xue Ma, Chuan Qin, Man Chen, Hai-Han Yu, Yun-Hui Chu, Ting-Jun Chen, Dale B. Bosco, Long-Jun Wu, Bi-Tao Bu, Wei Wang, Dai-Shi Tian

**Affiliations:** 1 Department of Neurology, Tongji Hospital, Tongji Medical College, Huazhong University of Science and Technology, Wuhan, 430030 People’s Republic of China; 2 Department of Neurology, Mayo Clinic, Rochester, MN 55905 USA

**Keywords:** Neuromyelitis optica spectrum disorder, Regulatory T cells, Inflammatory response, Microglia, Macrophage

## Abstract

**Background and purpose:**

Neuromyelitis optica spectrum disorder (NMOSD) is mainly an anti-aquaporin 4 (anti-AQP4) autoantibodies-mediated idiopathic inflammatory demyelinating disease of the central nervous system. Systemic and local inflammatory responses play a key role in the pathophysiology of NMOSD. However, the role of the crucial immunomodulators CD4^+^CD25^+^ forkhead box P3^+^ (Foxp3) regulatory T cells (Tregs) has not been investigated in NMOSD.

**Methods:**

Twenty-five patients with anti-AQP4-postive NMOSD undergoing an attack and 21 healthy controls (HCs) were enrolled. Frequencies of T cell subsets and Tregs in the peripheral blood were assessed by flow cytometry. Additionally, a model of NMOSD using purified immunoglobulin G from anti-AQP4-antibodies-positive patients with NMOSD and human complement injected into brain of female adult C57BL/6J mice was established. Infiltrated Tregs into NMOSD mouse brain lesions were analyzed by flow cytometry, histological sections, and real-time quantitative Polymerase Chain Reaction. Astrocyte loss, demyelination, and inflammatory response were also evaluated in our NMOSD mouse model. Finally, we examined the effects of both depletion and adoptive transfer of Tregs.

**Results:**

The percentage of Tregs, especially naïve Tregs, among total T cells in peripheral blood was significantly decreased in NMOSD patients at acute stage when compared to HCs. Within our animal model, the number and proportion of Tregs among CD4^+^ T cells were increased in the lesion of mice with NMOSD. Depletion of Tregs profoundly enhanced astrocyte loss and demyelination in these mice, while adoptive transfer of Tregs attenuated brain damage. Mechanistically, the absence of Tregs induced more macrophage infiltration, microglial activation, and T cells invasion, and modulated macrophages/microglia toward a classical activation phenotype, releasing more chemokines and pro-inflammatory cytokines. In contrast, Tregs transfer ameliorated immune cell infiltration in NMOSD mice, including macrophages, neutrophils, and T cells, and skewed macrophages and microglia towards an alternative activation phenotype, thereby decreasing the level of chemokines and pro-inflammatory cytokines.

**Conclusion:**

Tregs may be key immunomodulators ameliorating brain damage via dampening inflammatory response after NMOSD.

**Supplementary Information:**

The online version contains supplementary material available at 10.1186/s12974-021-02266-0.

## Introduction

Neuromyelitis optica spectrum disorder (NMOSD) is an autoimmune disease of the central nervous system that preferentially induces inflammatory demyelinating lesions in the optic nerve and spinal cord [1, 2]. The humoral immunity mechanism plays an essential role in the pathogenesis of NMOSD, as autoantibodies against aquaporin-4 (APQ4) immunoglobulin G (IgG) bind to astrocytes evoking antibody-dependent cell-mediated cytotoxicity and complement-dependent cytotoxicity [3, 4]. Besides, upregulation of serum or cerebrospinal fluid proinflammatory cytokines, systemic and local AQP4-specific T cells, activation of resident microglia, and lymphocyte infiltration into the brain substantially contribute to brain damage in NMOSD [5–12]. However, the endogenous counterregulatory immune responses limiting inflammatory damage during NMOSD lesion formation are poorly understood.

CD4^+^CD25^+^forkhead box P3^+^ (Foxp3) regulatory T cells (Tregs) play a central role in immune regulation via the secretion of immunosuppressive cytokines (e.g., interleukin (IL)-10, IL-35, transforming growth factor- β (TGF-β)), granzyme, and perforin, and via cell-contact dependent mechanisms [13, 14]. They can be derived from the thymus, and be induced from naïve T cells in the periphery in response to inflammation [14]. Tregs maintain immune homeostasis and tolerance by modulating the functions of effector T cells, macrophages, antigen-presenting cells, and natural killer cells [15, 16]. In several autoimmune diseases, including multiple sclerosis [17–19], myasthenia gravis [20, 21], systemic lupus erythematosus [22], and rheumatoid arthritis [23, 24], an abnormal number or defective function of Tregs were observed [25]. However, the role of Tregs in the pathogenesis of NMOSD has not been clearly elucidated. Here, our work uncovers the crucial impact of Tregs on demyelination, and their underlying neuroprotective function in NMOSD.

## Materials and methods

### Patient population

According to the 2015 diagnostic criteria for NMOSD, we retrospectively reviewed clinical data from patients with AQP4-IgG-positive NMOSD admitted to Tongji Hospital (Tongji Medical College, Huazhong University of Science and Technology) between 1 July 2015 and October 1 2020. Serum AQP4-IgG was confirmed by cell-based assay**.** Blood samples from 25 NMOSD patients at acute phase and 21 healthy controls (HCs) were analyzed via flow cytometry to characterize the percentage and number of CD3^+^CD19^−^ T cells, CD3^+^CD4^+^ T cells, CD3^+^CD8^+^ T cells, Tregs (CD3^+^CD4^+^CD25^+^CD127^low+^ T cells), effector Tregs (CD45RO^+^CD3^+^CD4^+^CD25^+^CD127^low+^ T cells), and naïve Tregs (CD45RA^+^CD3^+^CD4^+^CD25^+^CD127^low+^ T cells). All research procedures were approved by the Institutional Review Board at Tongji Hospital (institutional review board ID: TJ-IRB20190502) and written informed consents from all the participants were obtained.

### IgG purification

Serum was collected from 4 donors diagnosed with AQP4-IgG-positive NMOSD and 3 healthy controls. The clinical data of patients with NMOSD is shown in Supplementary Table S1. IgG was isolated with protein G-agarose and prepared as lyophilized powder as described [26]. The IgG in lyophilized powder was dissolved in phosphate-buffered saline (PBS) at pH 7.4 and sterile filtered. The final IgG concentrations were set at 20 mg/mL and termed as either NMOSD-IgG (from patients with NMOSD) or Con-IgG (Control-IgG, from healthy controls).

### NMOSD animal model procedure

The Committee on the Ethics of Animal Experiments of Tongji Medical College approved all animal experiments. Animal experiments were conducted in accordance with the National Institutes of Health Guide for the Care and Use of Laboratory Animals. For all experiments, 8–12 weeks old weight-matched female C57BL/6 mice were used. Mice were maintained in a suitable air-filtered environment and fed normal chow.

To induce NMOSD pathology, mice were first anesthetized with isoflurane (5% induction, 1.2–1.6% maintenance) and mounted onto a stereotactic frame (RWD Life Science, Shenzhen, China). Following a midline scalp incision, the bregma was exposed. For intracerebral injection, a 1-mm burr hole was made with a high-speed drill (RWD Life science) 2 mm to the right of the bregma. A 33-G needle attached to a 25-ul gas-tight glass syringe (Hamilton, Reno, NV, USA) was inserted 3 mm deep to infuse 6 uL NMOSD-IgG and 4 uL human complement (hC) (Innovative research, IPLA-CSER) (10 uL at 0.5 uL/min). The syringe was then removed and the scalp was closed using a 4-0 nylon suture.

### Immunofluorescence

Seven days after injection, mice were anesthetized and transcardially perfused with 15 mL ice-cold PBS, followed by 15 mL 4% paraformaldehyde in PBS. Brains were post-fixed overnight in 4% paraformaldehyde, and then immersed in gradient sucrose solutions for 72 h at 4 °C. Successive frozen sections were made (20-μm thickness) with a freezing microtome (Thermo Fisher Scientific) and prepared for immunostaining. Frozen sections were permeabilized with Immunostaining Permeabilization Solution with Triton X-100 (Beyotime, P0096), blocked with QuickBlock™ Blocking Buffer for Immunol Staining (Beyotime, P0260), and then immunostained with the following primary antibodies at 4 °C overnight: rabbit polyclonal anti-AQP4 (1:50, Proteintech), mouse anti-glial fibrillary acidic protein (GFAP) (1:200, Cell Signaling Technology), rat anti-myelin basic protein (MBP) (1:50, Millipore), rabbit polyclonal anti-ionized calcium binding adaptor molecule 1 (Iba1) (1:500, Wako), rabbit anti-CD45 (1:200, Servicebio), rabbit polyclonal anti-lymphocyte antigen 6G (Ly-6G) (1:200, Servicebio), rat anti-CD3 (1:200, Bio-rad), rat anti-CD68 (1:500, Bio-rad), rat anti-CD16 (1:50, BD Biosciences), goat anti-CD206 (1:100, R&D systems), rabbit anti-arginase-1 (Arg-1) (1:200, Santa Cruz Biotechnology), and rabbit anti-inducible Nitric-Oxide Synthase (iNOS) (1:200, Proteintech) followed by the appropriate Alexa Fluor conjugated secondary antibody (1:400, Invitrogen) for 1 h at room temperature. According to the methods in the previous study [26–28], the coronal brain slices through the needle tract were selected. Immunofluorescence was detected using either a fluorescent microscope (Olympus) or confocal laser scanning microscopy (Olympus). ImageJ (NIH) was used to measure the lesion area and cell counting.

### Depletion of Tregs

Mice were intraperitoneally injected with 300 mg of CD25-specific mAb (Bio X Cell, clone PC61) 48 h before NMOSD induction for in vivo depletion of Tregs as previously described [29].

### Adaptive transfer of Tregs

Briefly, Tregs were isolated from the spleens of C57BL/6 mice by magnetic cell sorting (Miltenyi Biotec). The purity was more than 85% as determined by flow cytometry. After isolation, approximately 800,000 CD4^+^CD25^+^ Tregs were intraperitoneally injected into mice immediately after NMOSD induction.

### Flow cytometry

Seven days after NMOSD induction, blood and spleens were collected. Peripheral blood mononuclear cells were separated with Ficoll (GE Healthcare) by density gradient centrifugation. Cells were then washed with PBS containing 0.5% bovine serum albumin (BSA) and prepared for staining. Spleens were pressed through a 70-μm cell strainer and then centrifuged at 300×*g* for 5 min. Red Blood Cell Lysis Buffer was added to the pellet (Beyotime) to remove red blood cells. Cells were washed with PBS containing 0.5% BSA and prepared for staining.

To determine percentages of Tregs in the brain, mice were transcardially perfused with ice-cold PBS and brains were then removed. Brain tissues were mechanically chopped into 1 mm^3^ fragments and then digested in 1 mg/mL collagenase D (Roche) and 0.5 mg/mL DNase I in RPMI 1640 at 37 °C for 45 min. Suspension was passed through a 70-μm cell strainer and then centrifuged at 1200 rpm for 10 min at 4 °C. The pellet was resuspended in 4 mL of 36% Percoll (GE Healthcare) and overlaid on top of 2 mL 63% Percoll. Percoll was diluted with Hanks’ balanced salt solution. The gradient was centrifuged at 2000 rpm for 40 min at 20 ± 2 °C. Cells from the top of tissue debris to 63% Percoll interface were collected into a new tube and washed twice with PBS containing 0.5% BSA.

All collected cells were stained with the following anti-mouse antibodies: PerCP/Cyanine5.5 anti-mouse CD45 (Biolegend, clone 30-F11), FITC anti-mouse CD4 (Biolegend, clone RM4-5), PE anti-mouse CD25 (Biolegend, clone PC61), and APC anti-mouse Foxp3 (ebioscience, clone FJK-16s). Cells were fixated and permeabilized with Transcription Factor Buffer Set (BD Biosciences) to stain intranuclear Foxp3.

### Reverse transcription-quantitative polymerase chain reaction (RT-qPCR)

Mice were *i.p.* injected with 200 μL 1% neutral red (NR) (Sigma-Aldrich) in PBS for brain lesion labeling, then anesthetized and transcardially perfused with PBS 2–3 h later [30]. The NR-labeled lesion was dissected and cells were lysed with Trizol (Invitrogen). Reverse transcription with HiScript III 1st Strand cDNA Synthesis Kit (Vazyme, China) was then performed. Following this, primers for glyceraldehyde-phosphate dehydrogenase (GAPDH), Foxp3, CD103, glucocorticoid-induced tumor necrosis factor receptor-related protein (GITR), IKAROS family zinc finger 2 (IKZF2), IKAROS family zinc finger 4 (IKZF4), C-C Motif Chemokine Ligand 1 (CCL1), C-C Motif Chemokine Ligand 5 (CCL5), C-C Motif Chemokine Ligand 2 (CCL2), tumor necrosis factor-α (TNF-α), IL-6, interferon-γ (IFN-γ), IL-1β, and IL-10 were analyzed by RT-qPCR with SYBR-Green assays (Vazyme, China). RT-qPCR runs in triplicate, with two technical replicates per sample. GAPDH was used for internal reference. The expression levels of target genes were analyzed on the basis of the 2−ΔΔCT method [31]. The primer sequences were shown in Supplementary Table S2.

### Transmission electron microscopy (TEM)

Mice were anesthetized with 6% chloral hydrate and cerebral hemispheres were removed. NMOSD lesions through the needle tract were cut into 1 mm^3^ sections and tissues were post-fixed with 2.5% glutaraldehyde and stored in 4 °C. Samples were made for routine electron microscopy observation and examined under an electron microscope (Tecnai) at 200 kV.

### Luxol fast blue (LFB) staining

Frozen brain slices were stepwise dehydrated with alcohol before immersion into 0.1% LFB (Servicebio) at 60 °C for 4–6 h. Tissue sections were rinsed and differentiated in 0.05% lithium carbonate for 30 s, followed by 70% alcohol for 30 s. Then slices were differentiated in 95% alcohol and 100% alcohol for 5 min each. Images were taken with a light microscope (Olympus). Demyelination lesions were evaluated by ImageJ (NIH).

### Western blot

NR-labeled lesions were dissected and homogenized in cell lysis buffer (Beyotime, China) supplemented with phosphatase inhibitors (Beyotime, China). Tissue lysates were centrifuged at 15,000×*g* for 15 min at 4 °C. Supernatant was collected and protein concentration was determined. Total protein (20–40 μg) from each sample was resolved on 10% sodium dodecyl sulfate-polyacrylamide gels and blotted to 0.45 μM nitrocellulose filter membranes (Merck Millipore). Then the membranes were blocked with 5% skim milk or 5% BSA for 1 h at room temperature and then incubated over night with the primary antibodies. Primary antibodies included those against: CD16 (1:500, 553142, BD Pharmingen), iNOS (1:1000, 18985-1-AP, Proteintech), CD206 (1:1000, AF2535, R&D systems), Arg-1 (1:1000, 16001-1-AP, Proteintech), TNF-α (1:500, 17590-1-AP, Proteintech), TGF-β1 (1:500, 21898-1-AP, Proteintech), signal transducers and activators of transcription 3 (STAT3) (1:1000, 10253-2-AP, Proteintech), phosphorylation-STAT3 (p-STAT3) (1:1000, 9145, Cell Signaling Technology), and β-actin (1:4000, GB11001, Servicebio). Membranes were washed three times with Tris-buffered saline with 0.05% Tween-20 and incubated for 1 h at room temperature with horseradish peroxidase-labeled anti-rabbit, anti-goat, or anti-rat secondary antibody (1:5000, Invitrogen). Target proteins were visualized with enhanced chemiluminescence reagents (Servicebio, China) and evaluated via a CCD camera (Tanon 4600).

### Statistical analysis

Values are presented as median (interquartile range) or mean ± standard deviation (SD). Comparisons between two groups were performed with two-tailed non-parametric Mann-Whitney *U* test. Comparison between multiple groups was performed with one-way analysis of variance followed by Dunnett’s post hoc test.

## Results

### The proportion of Tregs in peripheral blood was decreased in NMOSD patients at acute stage

**Fig. 1:**
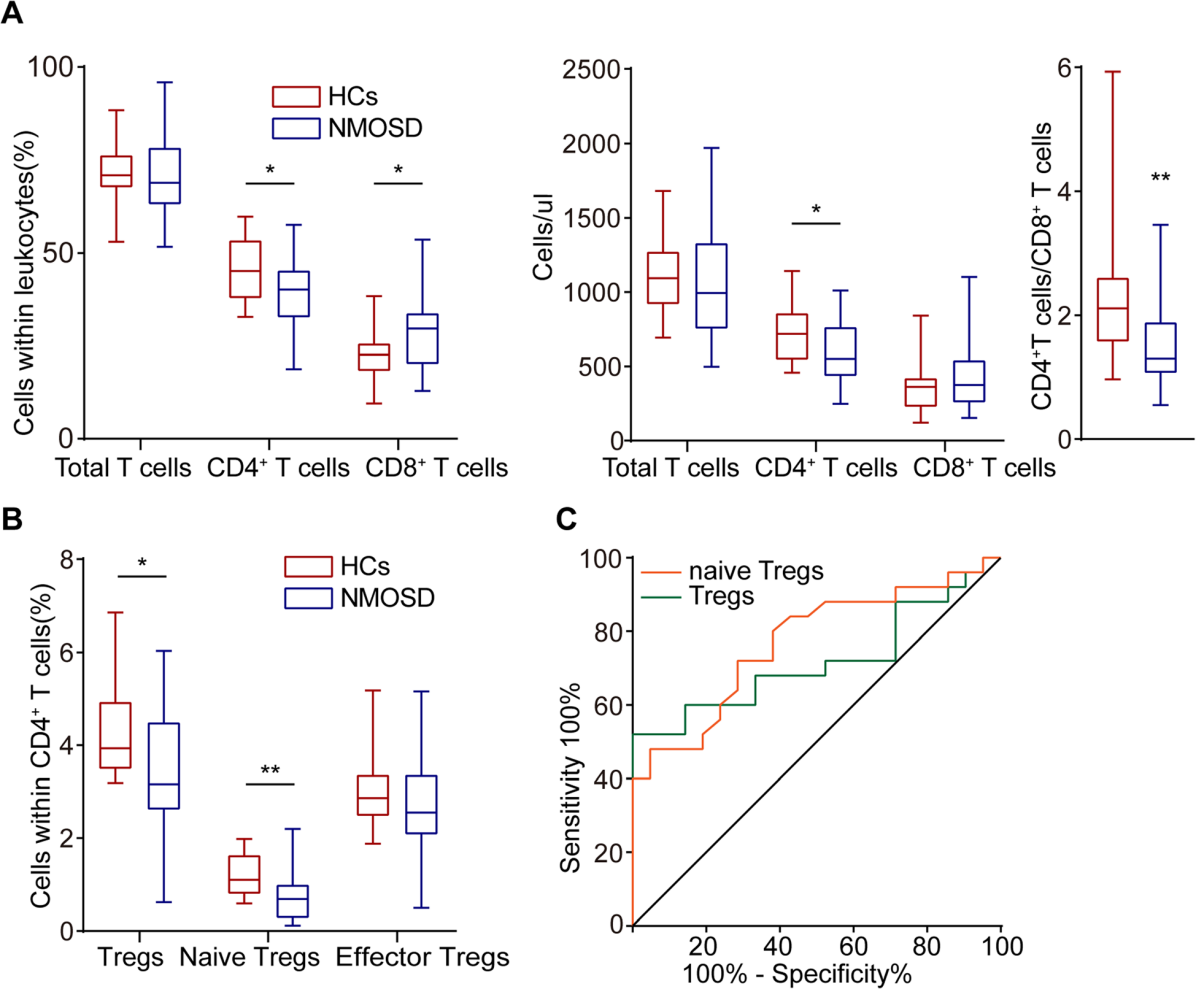
Reduced frequencies of Tregs in patients with NMOSD. **A** The proportions and counts per microliter of circulating CD3^+^CD19^−^ T cells, CD3^+^CD4^+^ cells, CD3^+^CD8^+^ cells out of CD3^+^CD19^−^ T cells in HC groups (*n* = 21) and NMOSD (*n* = 25). The ratio of CD3^+^CD4^+^ cells to CD3^+^CD8^+^ cells is shown in the box-plot diagram with median and interquartile range. *p < 0.05, **p < 0.01. **B** Percentages of Tregs (CD3^+^CD4^+^CD25^+^CD127^low+^ T cells), effector Tregs (CD45RO^+^CD3^+^CD4^+^CD25^+^CD127^low+^ T cells), and naïve Tregs (CD45RA^+^CD3^+^CD4^+^CD25^+^CD127^low+^ T cells) among the total CD3^+^CD4^+^ cells population from HC (*n* = 21) and NMOSD (*n* = 25) groups. *p < 0.05, **p < 0.01. **C** The percentage of Tregs and naïve Tregs in a receiver operating characteristic model with an area under the curve value of 0.718 and 0.770 respectively

Demographic and clinical features of included subjects are summarized in Supplementary Table S3. Age and gender distributions did not differ between NMOSD and HCs. The percentage of CD3^+^CD4^+^ T cells within the total T cell population and total number of CD3^+^CD4^+^ T cells were significantly reduced in patients with NMOSD, even though the total number of T cells did not differ between two groups. The percentage of CD3^+^ CD8^+^ T cells within the CD3^+^ T cells was statistically increased in NMOSD. A similar trend was observed in counts of CD3^+^CD8^+^ T cells; however, there was no statistical difference between the two groups. The ratio of CD3^+^CD4^+^ T cells to CD3^+^CD8^+^ T cells was consequently statistically decreased in NMOSD compared to HCs (Fig. 1A).

The percentages of Tregs and naïve Tregs within the CD3^+^CD4^+^ T cell population were also significantly lower in patients with NMOSD than that in HCs. However, there was no difference in the proportion of effector Tregs between two groups (Fig. 1B). We then analyzed Tregs and naïve Tregs in a receiver operating characteristic model. The area under curve for Tregs and naïve Tregs used to differentiate NMOSD from HC was 0.718 and 0.770 respectively, which were considered moderately predictive (Fig. 1C). These results indicate that Tregs, especially naïve Tregs, are greatly reduced in NMOSD patients at acute stage.

### Tregs accumulated in the brain in a mouse model of NMOSD

**Fig. 2:**
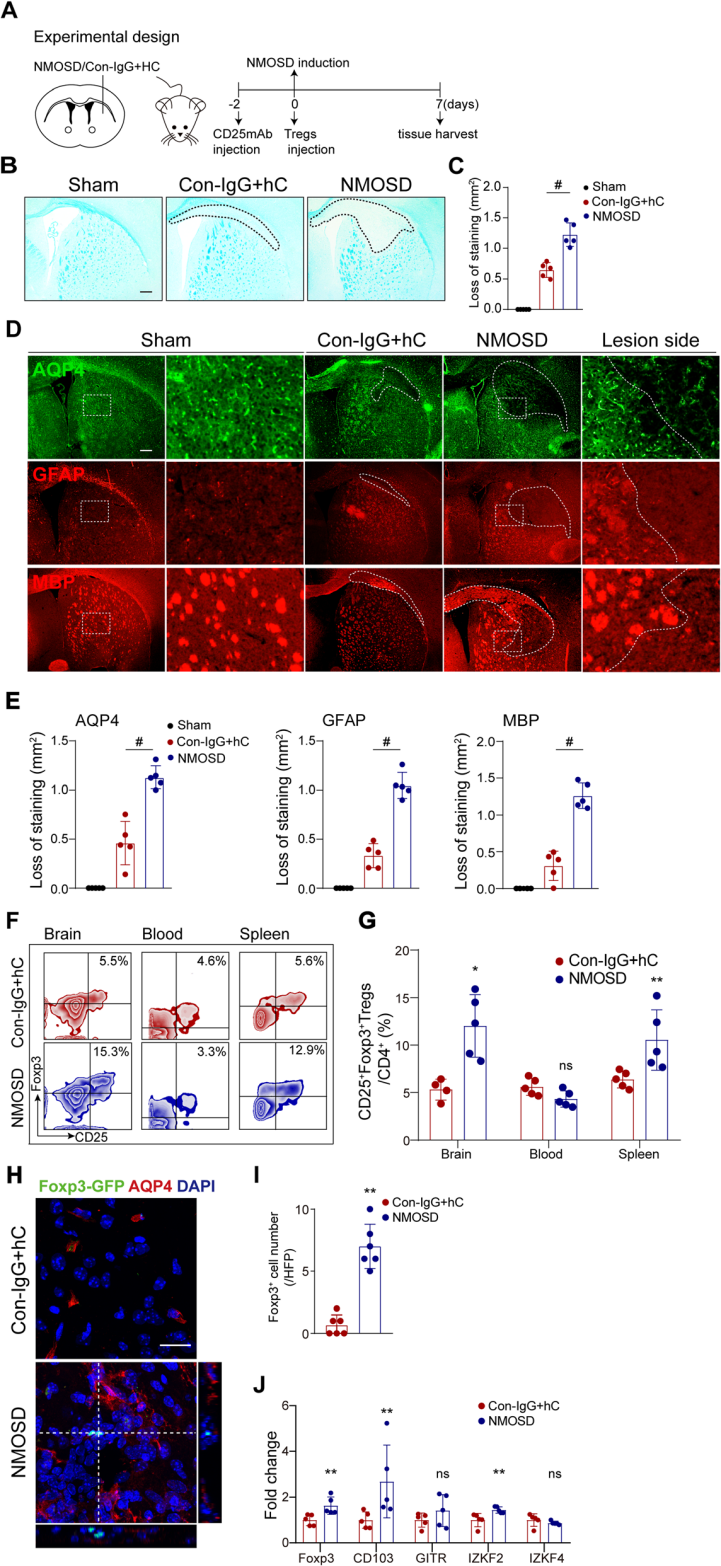
Tregs accumulated in the NMOSD-IgG and human complement-mediated brain lesion. **A** Schematic of experimental setup in mouse model of NMOSD. **B** Mice brains were injected with 6 μL NMOSD-IgG and 4 μL hC (NMOSD group) or Con-IgG and 4 μL hC (Con-IgG + hC). The demyelinated area was assessed by LFB staining 7 days post-injury. Scale bar, 300 μm. **C** Quantification of lesion size. Results displayed as mean and SD. *n* = 5 per group. #*p* < 0.0001 versus Con-IgG + hC group; statistical analyses were performed by one-way analysis of variance followed by Dunnett’s post hoc test. **D** Representative images of immunofluorescence staining for AQP4, GFAP, and MBP 7 days post-injection. Scale bar, 300 μm. **E** Quantifications of stained area. *n* = 5 per group; #*p* < 0.0001 versus Con-IgG + hC group; statistical analyses were performed by one-way analysis of variance followed by Dunnett’s post hoc test. **F** Flow cytometry zebra plots depicted percentages of CD4^+^CD25^+^Foxp3^+^ Tregs in the brain, blood, and spleen of representative NMOSD and control mice. **G** Quantification of Treg frequencies. *n* = 4–5 per group; *p < 0.05, **p < 0.01, ns, no significance, versus Con-IgG + hC group; statistical analyses were performed by Mann-Whitney *U* test. **H** Representative images of Foxp3-EGFP^+^ cells in brain slices of Foxp3-EGFP/cre mice after NMOSD induction. Scale bar, 20 μm. **I** Quantification of the number Foxp3-EGFP^+^ cells. *n* = 6 per group. **p < 0.01 versus Con-IgG + hC group; statistical analyses were performed by Mann-Whitney *U* test. **J** The mRNA expression of Tregs-related genes: Foxp3, CD103, GITR, IKZF2, and IKZF4 in NMOSD and control mice. *n* = 5 per group. *p < 0.05, **p < 0.01, ns, no significance, versus Con-IgG + hC group; statistical analyses were performed by two-tailed unpaired *t* test

Next, we investigate the role of Tregs in a mouse model of NMOSD. To this end, 6 μL NMOSD-IgG or Con-IgG plus 4 μL hC were intra-cerebrally injected into mice as previously described [26]. Areas of demyelination were evaluated by LFB staining 7 days post-injection. Non-stained areas were significantly larger in NMOSD-IgG and hC treated animals (NMOSD group) than those found in lesions induced by Con-IgG and hC (Fig. 2A–C). Similarly, loss of AQP4, GFAP, and MBP immunoreactivity was significantly greater in NMOSD group (Fig. 2D, E). These observations suggest that co-injection of NMOSD-IgG and hC produce key clinical histopathological features of NMOSD.

Seven days post-injection, we also found that the proportion of CD4^+^CD25^+^Foxp3^+^ Tregs in CD4^+^ T cells in the injured hemispheres and spleens of NMOSD group was largely increased. But there was no statistical significance in the proportion of Tregs in peripheral blood between the groups (Fig. 2f, g). To further confirm the infiltration of Tregs into brain lesions, we used Foxp3-enhanced green fluorescent protein (EGFP) mice within our model of NMOSD. We determined via confocal scanning imaging a significant increase of infiltrated Foxp3-EGFP-positive Tregs into the NMOSD lesion (Fig. 2h, i). Consistently, RT-qPCR results showed that relative expressions of Tregs signature genes, Foxp3, CD103, and IKZF2 but not IKZF4 and GITR, were significantly upregulated in NMOSD lesions when compared to controls group (Fig. 2j).

### NMOSD lesions were enhanced by Tregs depletion and ameliorated by adoptive transfer of Tregs

**Fig. 3:**
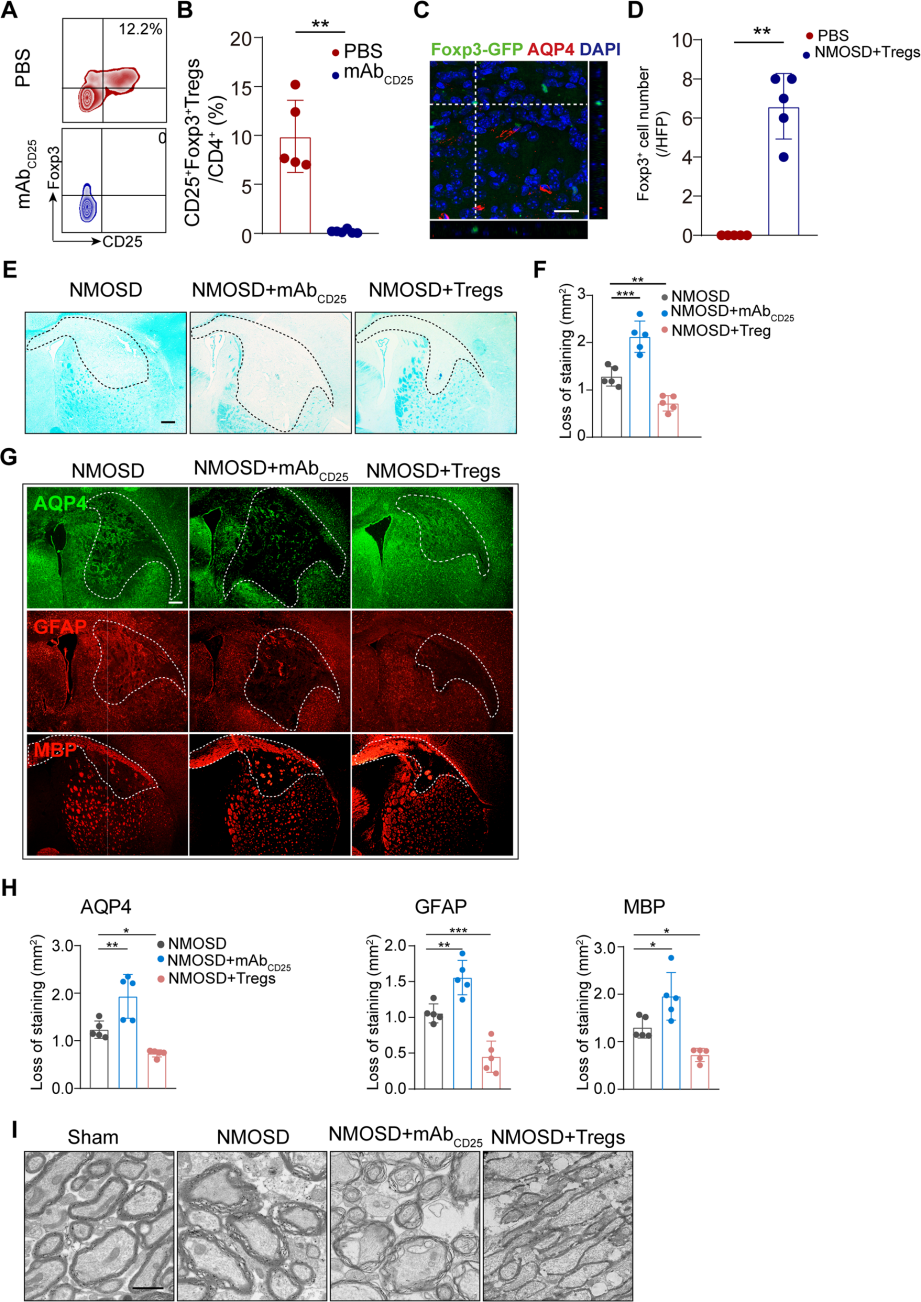
Tregs depletion aggravated NMOSD lesion development, while adoptive transfer of Tregs inhibited lesion progression. **A** Representative flow cytometric identification of anti-CD25mAb antibodies used to deplete CD4^+^CD25^+^Foxp3^+^ Tregs gated on CD4^+^ T cells in spleen at 9 days after injection. **B** Quantitative analysis of CD4^+^CD25^+^Foxp3^+^ Tregs out of CD4^+^ T cells. *n* = 5–6 per group. **p < 0.01 versus PBS group; statistical analyses were performed by Mann-Whitney *U* test. **C** Representative histological recognition of adoptively transferred Fxop3-EGFP^+^ Tregs in NMOSD lesions. Scale bar, 20 μm. **D** Quantification of the number of adoptively transferred Foxp3-EGFP^+^ cells. *n* = 5 per group. **p < 0.01 versus PBS group; statistical analyses were performed by Mann-Whitney *U* test. **E** Representative images of the white matter lesions by LFB staining. Scale bar, 300 μm. **F** Quantitative analysis of lesion size. *n* = 5 per group. **p < 0.01, ***p < 0.001, versus NMOSD group; statistical analyses were performed by one-way analysis of variance followed by Dunnett’s post hoc test. **G** Representative images of AQP4, GFAP, and MBP staining area in different groups. Scale bar, 80 μm. **H** Quantification of AQP4, GFAP, and MBP area of staining. *n* = 5 mice for each group. *p<0.05, **p < 0.01, ***p < 0.001, versus NMOSD group; statistical analyses were performed by one-way analysis of variance followed by Dunnett’s post hoc test. **I** Representative TEM images of myelination in the corpus callosum of mice. Scale bar, 1 μm

To address whether Tregs play a role in NMOSD lesion development, we first depleted Tregs with anti-CD25-specific antibodies. We observed approximately 99% depletion of CD4^+^CD25^+^Foxp3^+^ Tregs in the spleens of mice after anti-CD25-specific antibodies injection (Fig. 3A, B), which is consistent with the previous studies [29]. We also investigated the role of adoptive transferred Tregs. Foxp3-EGFP^+^ Tregs were isolated from spleens of Foxp3-EGFP mice and then injected into wild-type mice with NMOSD**.** Indeed, we observed a few Foxp3-EGFP^+^ cells in brain sections by confocal scanning imaging **(**Fig. 3C, D). Seven days after NMOSD induction, we evaluated histological changes via LFB staining and immunofluorescence. Mice lacking Tregs exhibited enlarged demyelinated areas, as well as loss of AQP4, GFAP, and MBP staining when compared to controls (Fig. 3E–G). By contrast, mice receiving Tregs displayed significantly less astrocyte loss and demyelination after NMOSD induction (Fig. 3E–G). Ultrastructural changes of myelination after NMOSD were assessed by TEM (Fig. 3I). Representative images of the corpus callosum of mice with NMOSD indicated vacuole or grid-liked changes. Disrupted myelin was substantially increased in Tregs-depleted mice, while a relatively integrated lamellar myelin sheath was observed in Tregs-transferred mouse. Altogether, Tregs appear to have a protective role in mitigating tissue damage in mice with NMOSD.

### Tregs depletion aggravated inflammatory infiltration after NMOSD, while adoptive transfer of Tregs suppressed inflammatory response

**Fig. 4:**
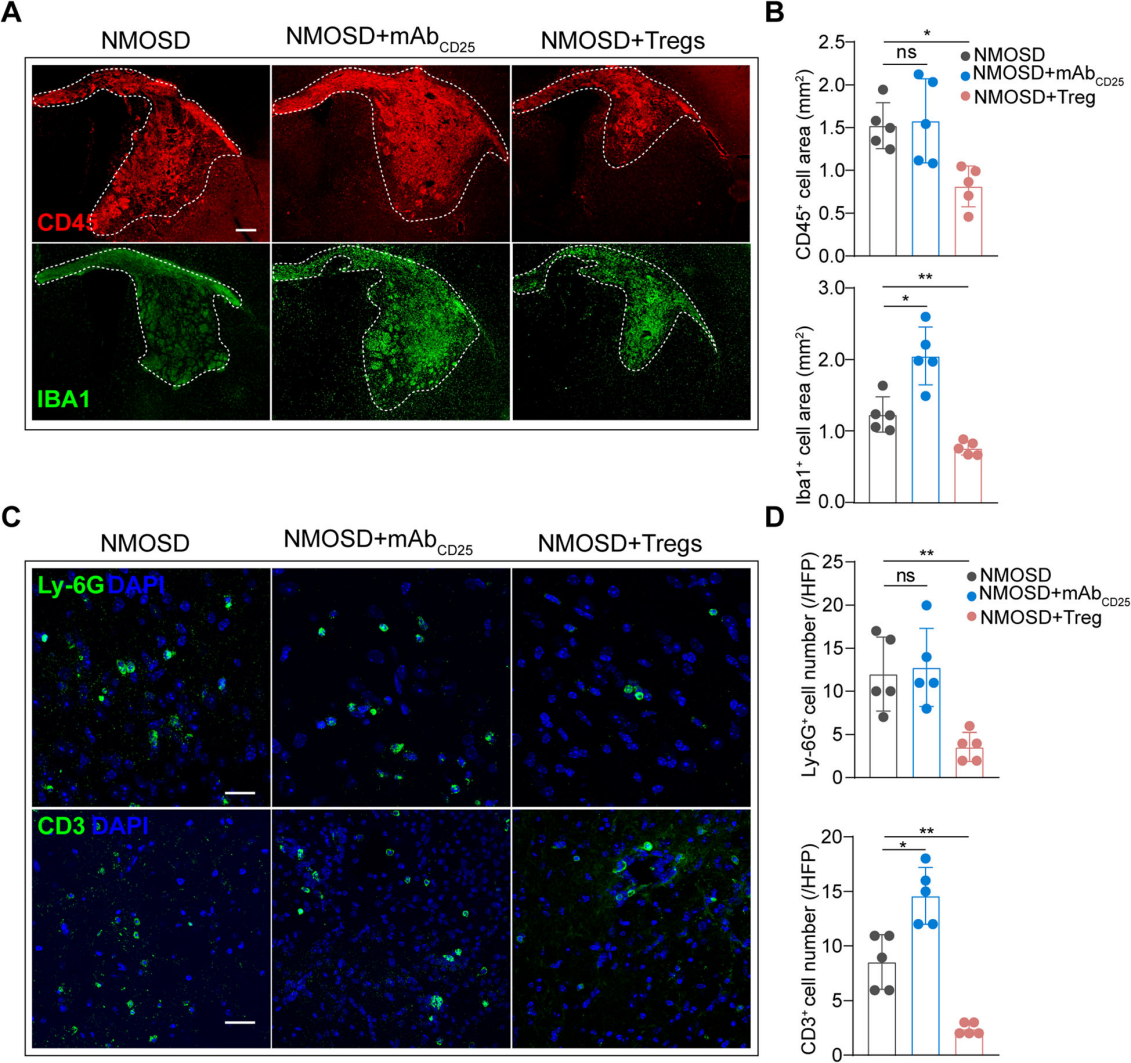
NMOSD-IgG and hC induced inflammatory activation and infiltration were partially enhanced by Tregs ablation but reversed by adoptive transfer of Tregs. **A** Representative images of CD45 and Iba1 staining. Scale bar, 300 μm. **B** Quantitative analysis of CD45 and Iba1 staining areas. *n* = 5 per group. *p < 0.05, **p < 0.01. ns, no significance, versus NMOSD group; statistical analyses were performed by one-way analysis of variance followed by Dunnett’s post hoc test. **C** Representative images of Ly-6G and CD3 staining. Scale bar, 300 μm. **D** Quantitative analysis of Ly-6G^+^ and CD3^+^ cell numbers. *n* = 5 per group. *p < 0.05, **p < 0.01. ns, no significance, versus NMOSD group; statistical analyses were performed by one-way analysis of variance followed by Dunnett’s post hoc test

Leukocyte activation and infiltration play a major role in the proinflammatory damage caused by NMOSD induction. To explore whether Tregs could affect inflammatory response to NMOSD lesions, we examined leukocyte subsets at the lesion sides via immunofluorescence staining. CD45^+^ leukocytes, Iba1^+^ macrophages/microglia, Ly-6G^+^ neutrophils, and CD3^+^ T cells were detected in mice following NMOSD induction. Adoptive transfer of Tregs largely decreased the invasion of CD45^+^ leukocytes (Fig. 4A, B). Further, supplementation of Tregs also markedly reduced the Iba1^+^ staining area within the NMOSD lesion, while depletion of Tregs increased Iba1^+^ area of staining (Fig. 4A, B). Neutrophil counts were also significantly lower in NMOSD mice receiving Tregs (Fig. 4C, D). CD3^+^ T cells were dramatically increased in Tregs-deficient mice and significantly reduced by injection of Tregs compared to mice with NMOSD (Fig. 4C, D). Collectively, our data indicate Tregs ameliorate inflammatory cells infiltration into NMOSD lesions.

### Tregs partially changed macrophages/microglia inflammatory status

Tregs, as suppressors of the adaptive immune system, are capable of steering macrophages/microglia differentiation towards alternatively activated macrophages [32]. Macrophage invasion and microglia activation are important pathologic features of NMOSD mouse models. Macrophages and microglia can be broadly categorized into two functional phenotypes, classical/pro-inflammatory, and alternative/anti-inflammatory. Pro-inflammatory phenotypes are characterized by upregulation of CD16 Fc receptors, CD32, iNOS, CD86, IL-1β, IL-23, and TNF-α. In contrast, anti-inflammatory phenotypes display up-regulated expression of CD206 and Arg-1, an increased production of IL-10 and TGF-β1.

**Fig. 5:**
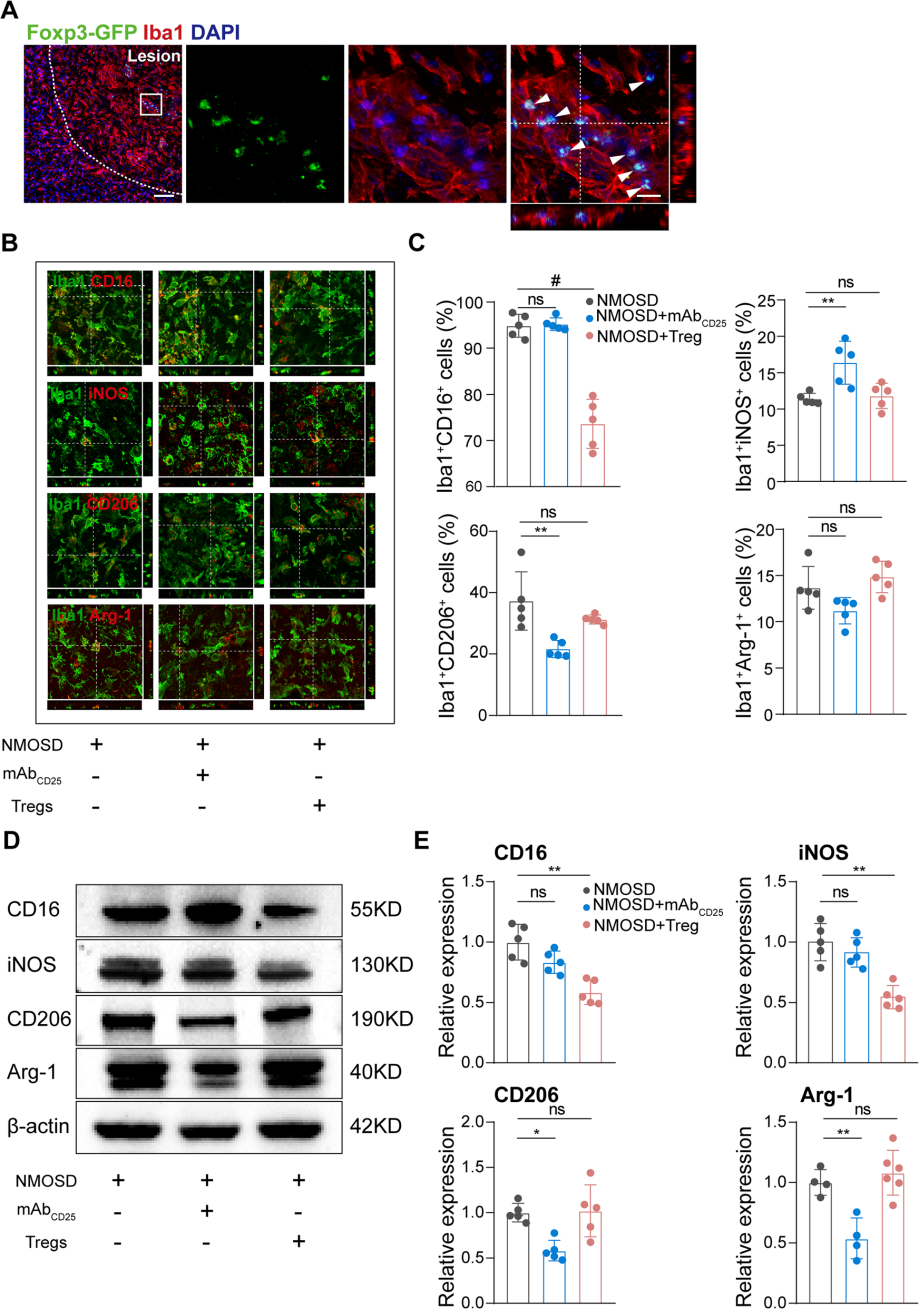
Tregs changed macrophages/microglia inflammatory status in NMOSD lesion. **A** Representative images of Iba1^+^ and Foxp3-EGFP^+^ cells in Foxp3-EGFP/cre mice with NMOSD. Scale bar, 80 μm. **B** Representative images of Iba1, CD16/32, iNOS, CD206, and Arg-1 stating 7 days post-NMOSD induction. Scale bar, 20 μm. **C** Percentages of CD16/32/iNOS/CD206/Arg-1-positive cells verse total Iba1-positive cells. *n* = 5 mice for each group. **p < 0.01, #*p* < 0.0001. ns, no significance, versus NMOSD group; statistical analyses were performed by one-way analysis of variance followed by Dunnett’s post hoc test. **D** Representative western blots of CD16/32/iNOS/CD206/Arg-1 in lesion samples. **E** Quantitative analysis of CD16, iNOS, CD206, and Arg-1 relative expression. *n* = 4–6 mice for each group. *p < 0.05, **p < 0.01. ns, no significance, versus NMOSD group; statistical analyses were performed by one-way analysis of variance followed by Dunnett’s post hoc test

To this end, we found that Tregs were predominantly detected and co-localized with Iba1^+^ macrophages and microglia in the lesion area of Foxp3-EGFP mice 7 days post-NMOSD induction (Fig. 5A), which revealed Tregs may closely interact with Iba1^+^ macrophages/microglia. To examine the modulation of Tregs on macrophages/microglia, we preformed double labeling of Iba-1 with pro-inflammatory markers (CD16/32 and iNOS) and anti-inflammatory markers (CD206 and Arg-1). The percentage of Iba1^+^CD16/32^+^ cells in the NMOSD lesion were largely unchanged in Treg-depleted mice, but was significantly reduced in mice receiving Tregs when compared to controls. Conversely, the proportion of Iba1^+^iNOS^+^ cells in Tregs-depleted mice was increased, while the proportion was largely similar in mice receiving supplemental Tregs when compared to controls. The absence of Tregs significantly reduced the proportion of Iba1^+^CD206^+^ cells compared to mice with NMOSD. There was no statistical significance in the percentage of Iba1^+^Arg-1^+^ cells on conditions of Tregs depletion or adoptive transfer of Tregs (Fig. 5B, C). Mostly consistent with immunostaining experiments, western blot results showed similar alterations at the protein level for pro- and anti-inflammatory markers. For example, classical activation markers (iNOS and CD16) were reduced by adoptive transfer of Tregs while alterative activation markers (CD206 and Arg-1) were decreased by Tregs depletion (Fig. 5D, E). These results indicate that Tregs shift macrophage/microglia towards an anti-inflammatory phenotype in our mouse model of NMOSD.

### Tregs reduced the expression of chemokines and pro-inflammatory cytokines

**Fig. 6:**
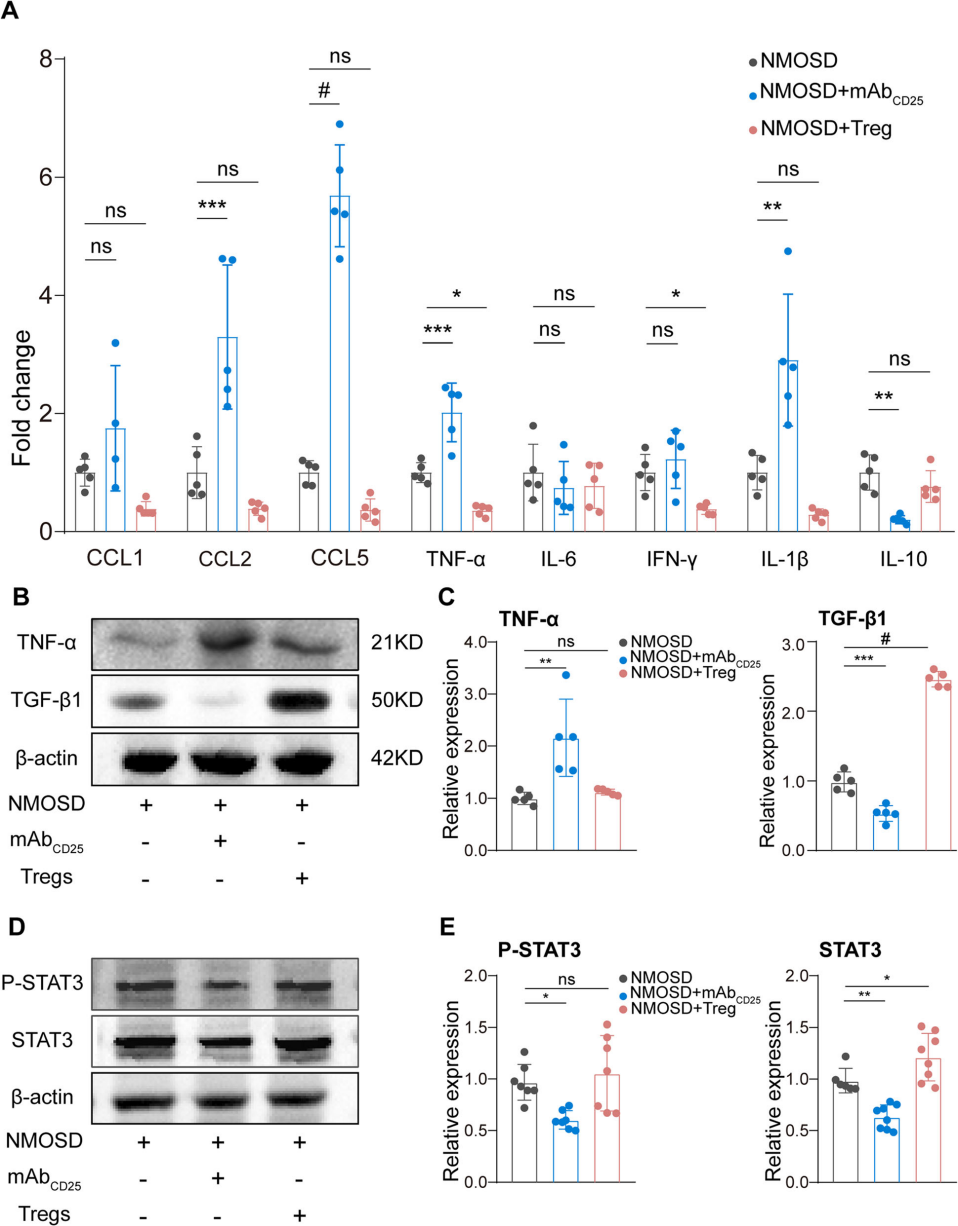
Tregs reduced the expression of chemokines and pro-inflammatory cytokines. **A** Quantitative RT-PCR analysis of CCL1, CCL2, CCL5, TNF-α, IL-6, IFN-γ, IL-1β, and IL-10 expression 7 days after NMOSD induction. *n* = 4–5 mice for each group. *p < 0.05, **p < 0.01, ***p < 0.001, #*p* < 0.0001. ns, no significance, versus NMOSD group; statistical analyses were performed by one-way analysis of variance followed by Dunnett’s post hoc test. **B** Representative western blots of TNF-α and TGF-β1. **C** Quantitative analysis of TNF-α and TGF-β1 expression. *n* = 5 mice for each group. *p < 0.05, **p < 0.01, ***p < 0.001, #*p* < 0.0001. ns, no significance, versus NMOSD group; statistical analyses were performed by one-way analysis of variance followed by Dunnett’s post hoc test. **D** Representative Western blots of total protein and phosphorylated STAT3 from the NMOSD lesion. **E** Quantitative analysis of p-STAT3 relative to β-actin and STAT3 relative to β-actin. *n* = 7–8 mice for each group. *p < 0.05, ***p < 0.001, ns, no significance, versus NMOSD group; statistical analyses were performed by one-way analysis of variance followed by Dunnett’s post hoc test

Chemokines and cytokines are essential factors for the development of NMOSD lesions [7]. Using RT-qPCR, we found that chemokines (CCL2, CCL5) and pro-inflammatory cytokines (TNF-α, IL-1β) were greatly increased by the depletion of Tregs, while the expression of anti-inflammatory cytokine IL-10 was significantly reduced. Conversely, chemokines, CCL1, CCL2, and CCL5 were tended to be decreased, and a significant reduction of pro-inflammatory factors, TNF-α and IFN-γ, was induced by Treg-adoptive transfer (Fig. 6A). Western blot results also confirmed that TNF-α was greatly increased at the protein level in Tregs-depleted mice. In addition, the expression of TGF-β1 was significantly reduced by Tregs depletion and increased by adoptive transfer of Tregs (Fig. 6B, C).

Tregs are capable of secreting immunosuppressive cytokines, including IL-10 and TGF-β, to exert their inflammation-dampening function [33]. To clarify which signaling pathways were involved, we performed western blots to evaluate the IL-10-related pathways. To this end, we evaluated the total protein expression and phosphorylation level of STAT3 that have been previously reported to be regulated by IL-10 for macrophage polarization [34, 35]. The relative levels of p-STAT3 and STAT3 were dramatically reduced by Treg depletion when compared to controls, but p-STAT3 levels did not change in the Tregs-transferred group (Fig. 6d, e). Though there is possibility that fewer STAT3^+^ cells are included in the sample after Treg depletion, our results suggest that Tregs may exhibit their inflammation-dampening function by inhibiting chemokines and secreting immunosuppressive cytokines to inhibit STAT3 pathway.

## Discussion

**Fig. 7:**
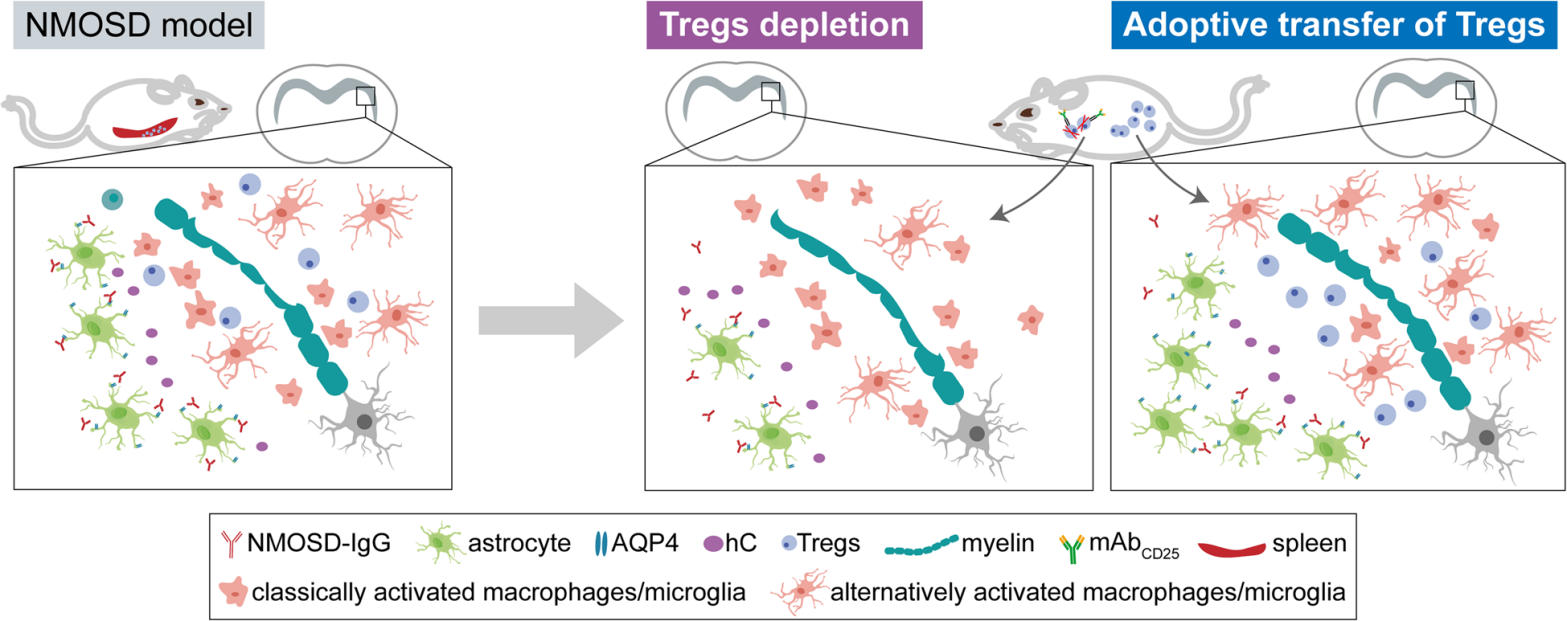
Schematic diagram shows that Tregs attenuate astrocyte loss and demyelination in NMOSD through modulating macrophages/microglia inflammatory status

It is well established that autoimmune inflammation response contributes substantially to the pathogenesis of NMOSD. Current research for NMOSD pathophysiology has mainly focused on humoral immune response, T cell-mediated pathogenesis, and proinflammatory cytokines [2, 9, 36–38]. Our study yielded new findings that the percentage of Tregs and naïve Tregs in peripheral blood were reduced in patients with NMOSD at acute phase. We also found in a mouse model of NMOSD, Tregs were enriched during brain lesion formation and were able to limit tissue damage through decreasing invasion of macrophages, neutrophils, and T cells, modulating macrophage/microglia inflammatory status, and reducing the level of chemokines and pro-inflammatory cytokines (Fig. 7).

Tregs play a critical role in the maintenance of peripheral immune tolerance and are composed of two types, naïve Tregs, developed in the thymus, and effector Tregs, derived from naïve CD4^+^ T cells in the periphery after antigenic stimulation [13]. Naïve Tregs are present in healthy individuals and exert their suppressive effects during normal surveillance of self-antigens [13]. There is a selective decline of naïve Tregs in several autoimmune diseases, such as type 1 diabetes and multiple sclerosis [39]. There are several contradictory reports on Treg cells in NMOSD: Tregs-related genes were found significantly decreased in NMOSD patients [33]. Other studies, on the other hand, reported that the frequency of Foxp3^+^ cells among CD4^+^ T cells did not differ between NMOSD and HCs [9, 40]. Our study, consistent with the former ones, showed a lower percentage of Tregs in patients with acute NMOSD when compared to HCs, particularly the naïve Tregs, suggesting that susceptibility to NMOSD is linked to deficiencies in naive Tregs. Further studies with larger sample size should be conducted to determine whether decreased Tregs number or percentage was a signature of NMOSD attacks.

In accordance with our findings in NMOSD patients, the percentage of Tregs in peripheral blood cells of mice following NMOSD induction tended to be lower. Notably, dramatically elevated percentages of Tregs in the spleen and brain were detected. One possibility was that spleen Tregs in NMOSD were expanded and then infiltrated into the brain NMOSD lesion through chemotactic effects. Nevertheless, the underlying mechanisms responsible for the decline of Tregs in peripheral blood need to be further elucidated.

Tregs attenuate brain injury in several CNS disease models, including acute experimental stroke [29, 41], experimental autoimmune encephalomyelitis [42], acute experimental traumatic brain injury [43], and intracerebral hemorrhage [44]. Although early injury mechanisms such as antibody-dependent cell-mediated cytotoxicity and complement-dependent cytotoxicity are more likely to determine the kinetics of astrocyte loss and demyelination in NMOSD, local, and systematic immune dysregulation are also involved, including inflammatory infiltration and dysregulated systematic immune response [45]. In our study, the effect of Tregs in brain lesions was evaluated in two independent experimental paradigms, anti-CD25 antibodies-mediated depletion and adoptive transfer of Tregs. Our data illustrated that Tregs significantly dampened microenvironment inflammation by reducing T cells and neutrophils infiltration and changing macrophages/microglia inflammatory status. These results are consistent with previous studies suggesting that Tregs exhibit a immunosuppressive effect by inhibiting other immune cells in disease models, including intracerebral hemorrhage [44, 46], traumatic brain injury [47], myocardial infarction [48], etc. However, the forward and backward feedback loops involved in the interaction between Tregs and other immune cells, especially the Tregs-macrophages/microglia interaction, remains unclear.

Our previous study demonstrated an unanticipated central role for microglia in NMOSD pathogenesis [12]. NMOSD-IgG could induce astrocytic production of complement C3 and microglial activation was secondary to C3aR signaling. Meanwhile, signaling via C3aR also has an integral role in suppressing the induction and function of Tregs [49]. Based on these data, it is possible that, this early complement component signaling, make a critical contribution to both the classical activation of microglia and inhibition of Tregs immunosuppressive function in NMOSD. Further studies are needed to identify whether complement components also drive Tregs-microglia interaction in this disease. Our present study, on the other hand, provided additional evidence suggesting that Tregs-mediated induction of alternatively activated microglia was partly through chemokine and cytokine secretion and possibly modulate STAT3 signaling of microglia in NMOSD lesions. Apart from that, Tregs could promote macrophage/microglia efferocytosis by a transcellar signaling pathway [16, 50], which may exert neuroprotective role via engulfment of myelin debris and apoptotic cells during the pathogenesis of NMOSD.

One limitation of our study is that only female mice were utilized in our model of NMOSD. Although the preponderance of females among NMOSD-IgG seropositive patients is as high as 9:1–10:1 [51], our results failed to illustrate the possible influence from sex difference in NMOSD. Secondly, our intracerebral injection model could duplicate histologically key features of patients with NMOSD [51]. However, systematic inflammation process of the disease, such as anti-AQP4 autoantibodies produced by B cells and pathologically autoactivate T cells, might be lacking. Another limitation is that although CD25-specific antibodies are quite effective at ablating Tregs by a phagocytosis-mediated mechanism [52], previous studies have shown that it may also impact other CD25-upregulated activated effector T cells [53]. As such, we cannot rule out the possibility that other CD25-positive cells are involved in the observed phenomenon.

## Conclusion

The present study provides evidence that Tregs impede NMOSD brain injury. Accordingly, we believe that a therapeutic strategy for NMOSD targeting Tregs is promising and merits further investigation.

## Supplementary Information

**Additional file 1 **: **Table S1**. Clinical characteristics of patients with NMOSD and healthy controls from whom serum samples were collected and purified IgG. **Table S2**. The primer sequences. **Table S3**. Demographics and baseline characteristics of patients with NMOSD and healthy controls.

## Data Availability

The datasets used and/or analyzed during the current study are available from the corresponding author on reasonable request.

## Abbreviations

Anti-AQP4: Anti-aquaporin 4
Arg-1: Arginase-1
BSA: Bovine serum albumin
CCL1: C-C motif chemokine ligand 1
CCL2: C-C motif chemokine ligand 2
CCL5: C-C motif chemokine ligand 5
CD: Cluster of differentiation
Con-IgG: Control-IgG
EGFP: Enhanced green fluorescent protein
Foxp3: Forkhead box P3
GFAP: Glial fibrillary acidic protein
GITR: Glucocorticoid-induced tumor necrosis factor receptor-related protein
GAPDH: Glyceraldehyde-phosphate dehydrogenase
HCs: Healthy controls
hC: Human complement
IKZF2: IKAROS family zinc finger 2
IKZF4: IKAROS family zinc finger 4
IgG: Immunoglobulin G
iNOS: Inducible nitric-oxide synthase
IFN-γ: Interferon-γ
IL: Interleukin
Iba1: Ionized calcium-binding adaptor molecule 1
LFB: Luxol fast blue
Ly-6G: Lymphocyte antigen 6G
MBP: Myelin basic protein
NMOSD: Neuromyelitis optica spectrum disorder
NR: Neutral red
p-STAT3: Phosphorylation-STAT3
Tregs: Regulatory T cells
RT-qPCR: Reverse transcription-quantitative polymerase chain reaction
STAT: Signal transducers and activators of transcription
SD: Standard deviation
TGF: Transforming growth factor
TEM: Transmission electron microscopy
TNF: Tumor necrosis factor
