# Supplementary material for: Regulatory T cells protect against brain damage by alleviating inflammatory response in neuromyelitis optica spectrum disorder
Source: J Neuroinflammation. 2021 Sep 15;18:201. doi: 10.1186/s12974-021-02266-0 (PMC8444427; doi:10.1186/s12974-021-02266-0)
Supplement: Supplementary file 1 — Additional file 1 : Table S1. Clinical characteristics of patients with NMOSD and healthy controls from whom serum samples were collected and purified IgG. Table S2. The primer sequences. Table S3. Demographics and baseline characteristics of patients with NMOSD and healthy controls. [file 12974_2021_2266_MOESM1_ESM.docx]

**Table S1. Clinical characteristics of patients with NMOSD and healthy controls from whom serum samples were collected and purified IgG.**

| Patient No. | Disease onset phenotype | Age(y)/Gender | Serum anti-AQP4 antibodies titer | Disease duration (months) | EDSS at collecting samples |
| --- | --- | --- | --- | --- | --- |
| NMOSD-1 | LETM | 19/F | 1:1000 | 36 | 1.0 |
| NMOSD-2 | LETM | 65/F | 1:320 | 6 | 2.0 |
| NMOSD-3 | ON | 52/F | 1:320 | 2 | 2.0 |
| NMOSD-4 | LETM | 46/F | 1:3200 | 3 | 1.5 |
| Healthy control-1 | NA | 26/F | NA | NA | NA |
| Healthy control-2 | NA | 30/F | NA | NA | NA |
| Healthy control-3 | NA | 27/F | NA | NA | NA |

EDSS, Expanded Disability Status Scale; F, female; LETM, longitudinally extensive transverse myelitis;

NA, not applicable; NMOSD, neuromyelitis optica spectrum disorder; No, number; ON, optic neuritis

**Table S2. The primer sequences**

| Primer | Forward primer | Reverse primer |
| --- | --- | --- |
| GAPDH | GGTTGTCTCCTGCGACTTCA | TGGTCCAGGGTTTCTTACTCC |
| Foxp3 | GAAGAGCCTGCCTTGGTACATTCG | TGTGAAGGTTCCAGTGCTGTTGC |
| CD103 | GCAGAGAACCACAGGACGAAGATC | TCCGATGCTGCTGCCAATGATG |
| GITR | GCCAGACGCTACAAGACTTGCC | CGTAACTCACCGCTCTCATACACC |
| IKZF2 | GAGGCTGGAGAGGACAAGGAGAG | GGCTAAGACAGTATGTGGCAGGTG |
| IKZF4 | CTCGCTCAGTGCCAACTCCATC | CCACTTCCATCGCAGTAGCCTAAG |
| TNF-α | ACGGCATGGATCTCAAAGAC | \| AGATAGCAAATCGGCTGACG \| \| --- \| |
| IL-1β | TGTCTTGGCCGAGGACTAAGG | \| TGGGCTGGACTGTTTCTAATGC \| \| --- \| |
| CCL1 | AAGATGGGCTCCTCCTGTCC | TGTTAGTTGAGGCGCAGCTT |
| CCL2 | TTAAAAACCTGGATCGGAACCAA | GCATTAGCTTCAGATTTACGGGT |
| CCL5 | GCTGCTTTGCCTACCTCTCC | TCGAGTGACAAACACGACTGC |
| IL-6 | GACAACCACGGCCTTCCCTACTTC | TCATTTCCACGATTTCCCAGAGA |
| IFN-γ | AACGCTACACACTGCATCTTGG | GACTTCAAAGAGTCTGAGG |
| IL-10 | AGCAAGGCAGTGGAGCAGGT | GGAACTGAGGTATCAGAGGTAA |

**Table S3. Demographics and baseline characteristics of patients with NMOSD and healthy controls**

| Items | Healthy controls (n=21) | NMOSD(n=25) | *P*-value |
| --- | --- | --- | --- |
| Age at onset, median (IQR) | 49(43-55) | 36(29-54) | 0.0841 |
| Female, n (%) | 17(80.95) | 22(85.7) | 0.6857 |
| Initial attack, n (%) | NA | 12(48%) | NA |
| Disease onset phenotype, n (%) | NA |  | NA |
| ON | NA | 6(24%) | NA |
| TETM | NA | 13(52%) | NA |
| ADEM or ADEM-like (including brainstem attacks) | NA | 5(20%) | NA |
| Simultaneous ON and TETM | NA | 0 | NA |
| ADEM or ADEM-like (including brainstem attacks) and TETM | NA | 1(4%) | NA |
| Disease duration (months), median (IQR) | NA | 9(0.67-84) | NA |
| EDSS at onset, median (IQR) | NA | 1.0(1.0-1.5) | NA |
| EDSS at collecting samples, median (IQR) | NA | 1.5(1.0-2.5) | NA |
| AQP4-IgG titer, median (IQR) | NA | 100(1:32-1:320) | NA |
| Attack times before collecting sample, median (IQR) | NA | 0(0-1) | NA |
| Combined with other autoimmune diseases, n (%) | NA | 7(28%) | NA |
| Standardized usage of immunosuppressants before collecting sample, n (%) | NA | 12(48%) | NA |

Data are shown as median (IQR) for continuous variables, and as percentages for categorical variables. n is the total number of patients with available data. p-values comparing NMOSD and healthy controls are from Mann-Whitney U test or χ² test.

ADEM, acute disseminated encephalomyelitis; AQP4-IgG, seropositive for IgG against aquaporin-4; EDSS, Expanded Disability Status Scale; IQR, interquartile range.NA, not applicable; NMOSD, neuromyelitis optica spectrum disorder; ON, optic neuritis; TETM, longitudinally extensive transverse myelitis;
